# Promotion of variant human mammary epithelial cell outgrowth by ionizing radiation: an agent-based model supported by in vitro studies

**DOI:** 10.1186/bcr2477

**Published:** 2010-02-10

**Authors:** Rituparna Mukhopadhyay, Sylvain V Costes, Alexey V Bazarov, William C Hines, Mary Helen Barcellos-Hoff, Paul Yaswen

**Affiliations:** 1 Life Sciences Division, Lawrence Berkeley National Laboratory, Berkeley, CA 94720 USA; 2 Department of Laboratory Medicine, University of California, San Francisco, CA 94143 USA; 3 Department of Radiation Oncology, New York University School of Medicine, New York, NY 10016 USA

## Abstract

**Introduction:**

Most human mammary epithelial cells (HMEC) cultured from histologically normal breast tissues enter a senescent state termed stasis after 5 to 20 population doublings. These senescent cells display increased size, contain senescence associated β-galactosidase activity, and express cyclin-dependent kinase inhibitor, p16^INK4A ^(CDKN2A; p16). However, HMEC grown in a serum-free medium, spontaneously yield, at low frequency, variant (v) HMEC that are capable of long-term growth and are susceptible to genomic instability. We investigated whether ionizing radiation, which increases breast cancer risk in women, affects the rate of vHMEC outgrowth.

**Methods:**

Pre-stasis HMEC cultures were exposed to 5 to 200 cGy of sparsely (X- or γ-rays) or densely (1 GeV/amu ^56^Fe) ionizing radiation. Proliferation (bromodeoxyuridine incorporation), senescence (senescence-associated β-galactosidase activity), and p16 expression were assayed in subcultured irradiated or unirradiated populations four to six weeks following radiation exposure, when patches of vHMEC became apparent. Long-term growth potential and p16 promoter methylation in subsequent passages were also monitored. Agent-based modeling, incorporating a simple set of rules and underlying assumptions, was used to simulate vHMEC outgrowth and evaluate mechanistic hypotheses.

**Results:**

Cultures derived from irradiated cells contained significantly more vHMEC, lacking senescence associated β-galactosidase or p16 expression, than cultures derived from unirradiated cells. As expected, post-stasis vHMEC cultures derived from both unirradiated and irradiated cells exhibited more extensive methylation of the p16 gene than pre-stasis HMEC cultures. However, the extent of methylation of individual CpG sites in vHMEC samples did not correlate with passage number or treatment. Exposure to sparsely or densely ionizing radiation elicited similar increases in the numbers of vHMEC compared to unirradiated controls. Agent-based modeling indicated that radiation-induced premature senescence of normal HMEC most likely accelerated vHMEC outgrowth through alleviation of spatial constraints. Subsequent experiments using defined co-cultures of vHMEC and senescent cells supported this mechanism.

**Conclusions:**

Our studies indicate that ionizing radiation can promote the outgrowth of epigenetically altered cells with pre-malignant potential.

## Introduction

Carcinogenic consequences of radiation exposure have historically been attributed to targeted effects - misrepaired DNA damage directly caused by dose-dependent ionization events in the cell of cancer origin. Radiation can also induce *non-targeted *effects - altered cytokines and signaling that affect the cellular composition and microenvironment of irradiated tissues [1], and non-mutational, but heritable changes that alter cell-cell interactions and induce persistent phenotypes associated with malignant progression [2-4]. The potential carcinogenic contribution of these non-targeted effects, which are typically not linearly proportional to radiation dose, has not been well studied, particularly in primary human epithelial cells.

In this study, we used primary cultures of human mammary epithelial cells (HMEC) as an experimental system to directly evaluate the potential of ionizing radiation to promote the outgrowth of cells bearing a pre-malignancy-associated epigenetic change. In serum-free growth medium, HMEC from histologically normal breast tissues arrest growth after 5 to 20 population doublings, exhibit senescent morphologies, and express p16^INK4A ^(CDKN2A; p16) [5,6]. This p16-dependent form of senescence, termed *stasis*, is distinguished by irreversible growth arrest with 2N DNA content (reviewed in [7]). Stasis is associated with poorly defined imbalances in signal transduction brought on by cell culture conditions or oncogene activation, but is not directly associated with DNA damage or dysfunctional telomeres [8,9]. Stasis requires activation of another well-known tumor suppressor, pRB, which functions downstream of p16, and serves as a block to indefinite proliferation (immortality) - a prerequisite for malignant transformation. The heterogeneous p16 expression observed in human breast epithelial cells *in situ *[10], and frequent aberrations in the p16-pRB pathway in human tumors [11], suggest that conditions that influence its expression and silencing have physiological and pathological relevance.

HMEC cultured in a serum-free medium spontaneously yield rare variant (vHMEC) cells in which p16 genes are methylated and silenced at frequencies that differ among normal specimens from women [12]. In previous studies, such vHMEC have been shown to be susceptible to genomic instability associated with telomere and centrosome dysfunction [6,13,14]. In some cases following carcinogen or oncogene exposure, these vHMEC give rise to immortalized clones bearing chromosomal aberrations commonly observed in primary human breast tumors [15]. Here, we sought to determine whether low to moderate doses of radiation (5-200 cGy) would alter the rate of vHMEC outgrowth from non-malignant tissues. In replicate experiments using HMEC from four different specimens, we found that radiation caused shorter growth plateaus and significantly increased the rate at which p16(-) vHMEC grew out in long term cultures. Computer simulations using an agent-based model suggested that radiation accelerated selection of pre-existing vHMEC, a prediction that was confirmed experimentally. Thus non-targeted radiation effects can lower a critical cancer barrier by altering cell interactions to promote outgrowth of cells with pre-malignant phenotypes.

## Materials and methods

### Cell culture

Histologically normal breast tissues, obtained from surgically discarded reduction mammoplasty (B1400 and N17) and prophylactic mastectomy (B1389 & B1450) specimens with patients' informed consent and institutional review board approval, were provided by the UCSF Cancer Center and the Cooperative Human Tissue Network. Specimens B1400 and N17 were derived from disease-free women aged 50 and 17 years, respectively. Specimen B1389 was derived from a 53-year old woman with a family history of breast and ovarian cancer, diagnosed with non-proliferative fibrocystic disease. Specimen B1450 was derived from a woman of unspecified age, diagnosed with invasive ductal and lobular carcinoma in the contralateral breast. Tissue samples were minced and enzymatically dissociated using 0.1% w/v collagenase I in Dulbecco's Modified Eagle Medium at 37°C for 12 to 18 hrs. Small tissue fragments (organoids) remaining after digestion, were collected by centrifugation at 100 × g for two minutes. These organoids were stored frozen or seeded directly into Mammary Epithelial Cell Growth Medium (MEGM; Lonza, Walkersville, MD, USA). The resulting HMEC were cultured in serum-free MEGM medium as previously described [16] and verified to be mycoplasma-free (Bionique Testing Laboratories (Saranac Lake, NY, USA). HMEC were routinely subcultured when 80% confluent and reseeded at a densities of 5 × 10^3 ^cells/cm^2^. Total population doublings were estimated using the equation, Population Doublings (PD) = log (A/B)/log2, where A was the number of collected cells and B was the number of plated cells. The effects of plating efficiencies were not taken into account in these estimates, and the calculated values were therefore lower than actual values. In some experiments, 5-aza-2'-deoxycytidine was added at a concentration of 3.3 μM to the culture medium for 72 to 96 hours prior to harvest.

### Experimental design

Passage 4 HMEC were routinely seeded at a density of 0.25 × 10^6 ^cells per T-25 flask. The cells were grown for seven to eight days to 60% confluence prior to radiation exposure. Control plates were sham-irradiated. After irradiation, the cells were allowed to recover for 48 hrs, then dissociated with trypsin and replated at densities of 0.25 × 10^6 ^cells per 100 mm dish. The cultures were then incubated for four to six weeks. When visual inspection indicated that the largest patches of vHMEC had attained diameters of 1.3 to 2.5 cm, all the cultures were harvested for analysis. To generate growth curves, both irradiated and unirradiated HMEC cultures were maintained in triplicate through Passage 9.

### Irradiation

Low linear energy transfer (LET) exposures were conducted using either 160 kVp X-ray or ^137^Cs γ-irradiation. Cultures were exposed to single doses of 5, 25, 50, 100 or 200 cGy at dose rates of either 22.5 cGy/minute (5, 25, or 50 cGy) or 1 Gy/minute (100 or 200 cGy). High-LET radiation exposures were conducted using a 1 GeV/amu ^56^Fe ion source at the NASA Space Radiation Laboratory at Brookhaven National Laboratory (Upton, NY, USA).

### Senescence associated β-galactosidase (SA-βgal) activity

For visualization, cells were washed, fixed, and incubated overnight at 37°C with X-gal chromogenic substrate at pH 6.0 as described [17]. The cells were viewed and photographed using a phase contrast Nikon Eclipse TS100 microscope (Nikon Instruments Inc., Melville, NY, USA). For quantitation of SA-βgal (+) and (-) cells by flow cytometry, fluorescein digalactoside (FDG; Molecular Probes, Eugene, OR, USA) was used as a substrate as described [18-20]. Cells were dissociated and resuspended in phosphate buffer saline containing 5% fetal bovine serum (PBS/FBS). Diluted FDG solution was mixed with an equal volume of cell suspension, incubated at room temperature for three minutes and quenched by adding 10 volumes of PBS/FBS. Propidium iodide (1.25 μg/ml, Molecular Probes, Eugene, OR, USA) was added to exclude dead cells from analysis. Flow cytometry was performed using a FACScan flow cytometer and Cell Quest Pro software (Becton Dickinson, Franklin Lakes, NJ, USA). Determinations of the relative sensitivities of HMEC and vHMEC to radiation-induced senescence were estimated using the formula: , in which Ps(0) and Ps(D) were the respective percentages of SA-βgal(+) cells in a population before and after exposure to a radiation dose (D). The formula included the factor 1-Ps(0) to correct for SA-βgal(+) cells pre-existing in the population.

### p16 immunohistochemistry and immunofluorescence

Cells were fixed with 4% paraformaldehyde for 30 minutes, washed and permeabilized with 0.1% Triton: 3% H_2_O_2 _for five minutes, incubated with a 1:300 dilution of primary antibodies (JC2, Neomarkers, Fremont, CA, USA), and washed three to four times. For visualization, the cells were incubated with VectaStain ABC reagent, followed by DAB substrate (Vector Laboratories, Burlingame, CA, USA). For quantitation, the cells were incubated with Alexa Fluor 488 conjugated goat anti-mouse IgG (1:200) secondary antibodies. Nuclei were counterstained with 0.5 ng/ml 4,6-diamidino-2-phenylindole (DAPI; Molecular Probes, Eugene, OR, USA). Images for individual channels were acquired and quantitated using a Cellomics Array Scan V^TI ^(Thermo Scientific, Pittsburgh, PA, USA), and merged using Adobe Photoshop 6.0 software (Adobe Systems, San Jose, CA, USA).

### DNA synthesis

Cells were incubated for the final 24 hr prior to harvest with 10 μM bromodeoxyuridine (BrdU; BD Biosciences, San Jose, CA, USA), then dissociated, washed with medium containing 2% FCS, and fixed with 95% ethanol. Nuclei were prepared by incubating the fixed cells with 0.8% pepsin for 20 minutes at 37°C, followed by an IFA buffer (10 mM HEPES, 150 mM NaCl, 4% FBS, and 0.1% sodium azide) wash containing 0.5% Tween. The nuclei were then incubated with a 1:5 dilution of fluoroscein-conjugated anti-BrdU antibodies in IFA (BD Biosciences, San Jose, CA, USA) on ice for 30 minutes, followed by treatment with RNase A (5 μg/ml) and propidium iodide (5 μg/ml). Flow cytometry was performed as described above.

### Bisulfite sequencing

Genomic DNA was isolated from cells using the Wizard Genomic DNA Isolation kit (Promega, Madison, WI, USA). A total of 500 to 1,000 ng of DNA was treated with bisulfite using the EZ DNA Methylation-Gold kit (Zymo Research, Orange, CA, USA). Polymerase chain reaction amplification of the p16 gene promoter region was performed using the primer set 5' TTT TTA GAG GAT TTG AGG GAT AGG 3' (-159 to -136) and 5' CTA CCT AAT TCC AAT TCC CCT ACA 3' (+209 to +233) [21,22] and the following conditions: 95°C/two minutes × one cycle; 96°C/20 sec, 60°C/20 sec and 72°C/90 sec × 40 cycles; 72°C/five minutes × one cycle. PCR products were purified with the QIAquick gel extraction kit (Qiagen, Valencia, CA, USA) and cloned in the pGEMT vector (Promega, Madison, WI, USA). Individual clones were sequenced with M13 forward and/or reverse primers.

### Immunoblot analyses

Total cell lysates were prepared in SDS/Tris buffer with protease inhibitors (Complete, Roche Applied Science, Indianapolis, IN, USA) and 20 to 30 μg/lane were separated on gradient SDS-PAGE gels (Invitrogen). After transfer, the separated proteins were incubated with a 1:1000 dilution of p16 antibodies (JC2, Neomarkers), followed by imaging using an Odyssey infrared imaging system (Licor Biosciences, Lincoln, Nebraska, USA).

### Statistical analyses

Graphpad Prism 5 (GraphPad Software, Inc., La Jolla, CA, USA) and JMP IN 3 (JMP, Cary, NC, USA) software were used for all the statistical analyses.

### Agent-based modeling

Simulations were performed using Matlab software (The MathWorks, Natick, MA, USA). Agents were dispersed randomly *in silico *to simulate plating onto a 400 × 400 grid (1.5 mm/pixel) at initial densities of 1,200 agents/grid (20% confluence). The following rules were used for modeling the growth of HMEC: 1) agents could divide as long as there was open space surrounding them; 2) if there was no more open space, agents could still divide but would be *compressed *until they reached a minimum size; 3) when an agent had reached the maximum number of divisions allowed, it would stop dividing and its type changed permanently to a senescent type; 4) senescent agents could not reattach after detachment during subculture. The program was reinitiated using the resulting agents when the grid reached 80% saturation (approximately 6,000 agents/grid) to simulate re-plating. For each set of experimental conditions, five independent simulations were performed, leading to an average behavior with a relative standard error less than 10%. By representing the grid as a tensor whose vectors are properties of the included agents, the progression of the tensor could be visualized using the advanced imaging platform DIPimage (Image Processing Toolbox for Matlab, Delft University of Technology, Delft, The Netherlands). A growth plateau was defined as the period during which the rate of population doubling deviated significantly from an exponential rate. Plateau width was determined experimentally as the time to 80% confluence of HMEC cultures in which the majority of cells displayed morphological features of senescence immediately after plating.

## Results

### The progeny of HMEC exposed to a moderate radiation dose form larger, more numerous patches of SA-βgal (-) and p16 (-) cells

**Figure 1:**
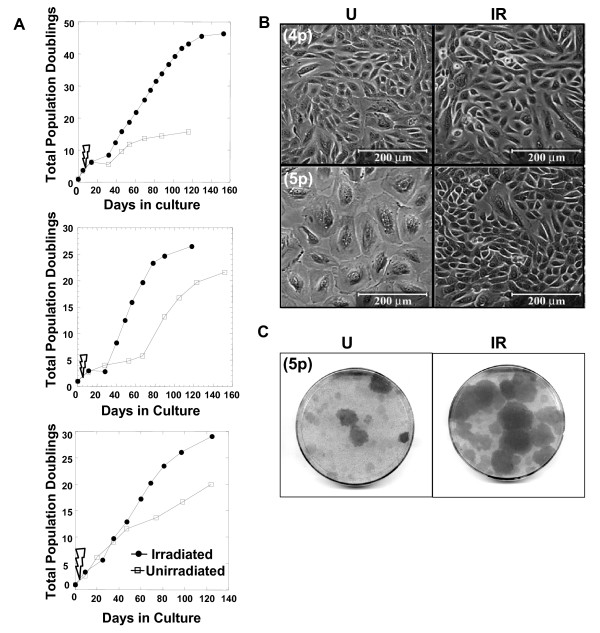
**The growth of human mammary epithelial cells cultures exposed to 200 cGy exceeds that of unirradiated cultures**. **(A) **Plots of the total population doublings of irradiated and unirradiated human mammary epithelial cells (HMEC) cultures versus days in culture for three experiments in which HMEC from different individuals were used (top to bottom; specimens B1450, B1400, and B1389). Each of the experimental points plotted in the graphs represents one passage. The time of irradiation between Passages 4 and 5 is indicated with a thunderbolt symbol. **(B) **Representative fields are seen in phase micrographs of unirradiated (U) and irradiated (IR) cultures at fourth passage (*4p*) 48 hrs after irradiation, or at fifth passage (*5p*) four to six weeks after irradiation. **(C) **Representative images of *5p *plates stained with crystal violet illustrate the presence of larger and more frequent patches of actively growing cells in cultures derived from irradiated vs. unirradiated cells.

We cultured primary HMEC from four histologically normal breast tissue specimens and grew them in serum-free MEGM medium under standard adherent conditions [5], subdividing and passaging them before they achieved confluence. At the fourth passage (*4p*) following establishment in culture, replicate HMEC cultures were exposed to a single dose of X-rays (0 or 200 cGy). The cells were then re-fed with complete growth medium and allowed to recover for two to four days prior to re-plating at identical cell densities, before control cultures reached confluence. Although the kinetics differed among specimens (B1450, B1400 and B1389), the irradiated cultures ultimately grew faster and underwent more population doublings than the unirradiated cultures in all cases (Figure 1A).

Soluble factors induced by ionizing radiation, such as TGFβ, affect the growth of unirradiated cells [2]. To determine whether the effect of radiation observed on the outgrowth of surviving cells was due to secreted factors, conditioned medium from irradiated or unirradiated cultures was added to the growth medium of unirradiated cultures. One set of irradiated and three sets of unirradiated B1400 or B1450 cultures were maintained separately. One unirradiated set served as a control and received only fresh medium at each feeding. A second unirradiated set received a 1:1 mixture of conditioned medium from irradiated cultures and fresh medium, while a third unirradiated set received a 1:1 mixture of conditioned medium from unirradiated cultures and fresh medium. Four replicates for each culture condition were maintained in this manner for four to six weeks. We did not observe any significant differences in the outgrowth of vHMEC in cultures supplemented with conditioned medium from irradiated or unirradiated cultures (data not shown). Thus soluble factors were not a likely cause of the differences observed.

We examined unirradiated and irradiated cultures 48 hrs following irradiation at *4p*. The cultures exhibited similar cell densities and morphologies; areas of small proliferative cells were interspersed with large, flat non-mitotic cells in both (Figure 1B, upper panels). However, four to six weeks after irradiation, large uniform patches of small proliferative cells were evident earlier and were more numerous in the subcultured (*5p*) irradiated populations than in the unirradiated populations (Figure 1B, lower panels, and Figure 1C).

**Figure 2:**
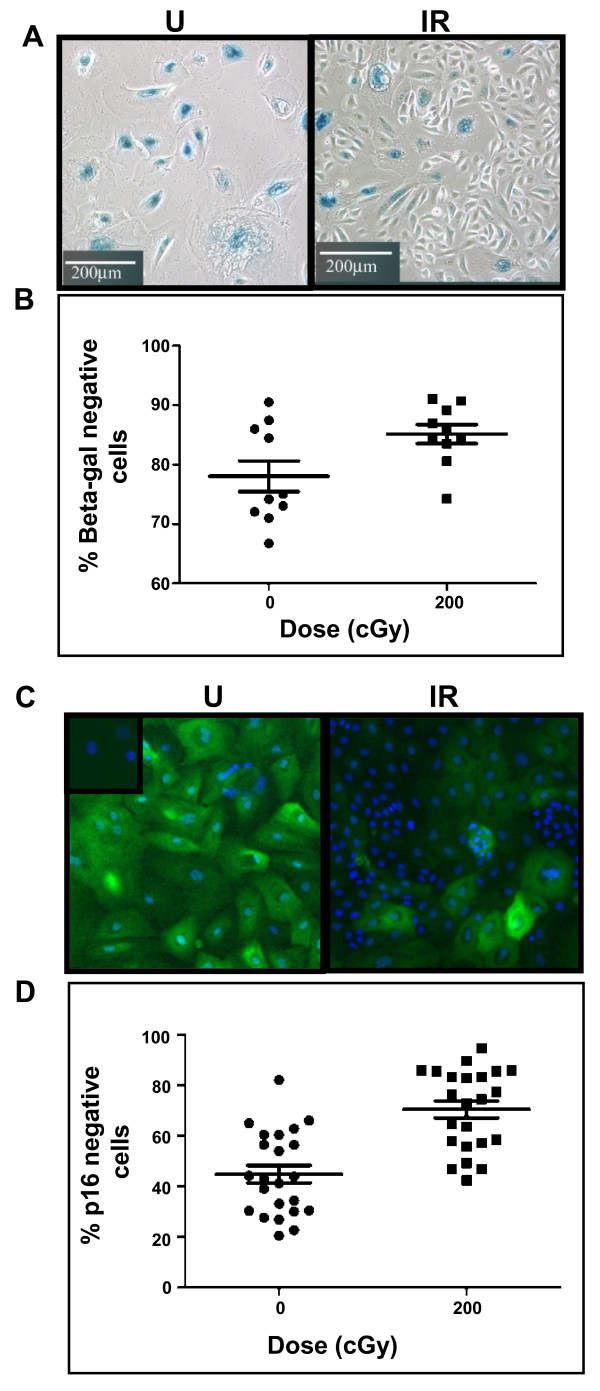
**Cultures derived from irradiated human mammary epithelial cells show more SA-βgal (-) and p16 (-) cells than those derived from unirradiated human mammary epithelial cells**. Representative fields are shown stained for **(A) **SA-βgal activity (blue reaction product) or **(C) **total p16 protein (green immunofluorescence). In (C), cell nuclei were counterstained with DAPI (blue). Inset = minus 1°Ab control. Note the greater presence of SA-βgal (-) and p16 (-) cells in the irradiated cultures. In **(B) **and **(D)**, cytometry was used to quantitatively compare the percentages of positively and negatively staining cells in the corresponding cultures. In (B), flow cytometry results of an experiment performed using B1450 cultures are summarized in a mean ± SE scatter plot (*P *< 0.001, non-parametric Wilcoxon test). In (D), the percentages of p16 (-) cells in unirradiated and irradiated B1400 and B1450 cultures were determined using a Cellomics Array Scan VTI automated fluorescence microscopic imaging system. For each condition, staining was measured for 460 fields per well and summarized in a mean ± SE scatter plot (*P *< 0.0001, non-parametric Wilcoxon test).

The expression of SA-βgal activity [17], a marker of cellular senescence, was measured in unirradiated and irradiated cultures when patches of vHMEC in unirradiated cultures reached 1.2 to 1.7 cm in diameter, typically four to six weeks post-irradiation. SA-βgal (-) cells were more abundant in irradiated than in the unirradiated cultures at these times (Figure 2A). To determine whether the differences observed between the percentages of SA-βgal (-) and (+) cells in irradiated and unirradiated cultures were statistically significant, 10 irradiated replicate cultures and 10 control replicate cultures from specimen B1450 were tracked independently. Flow cytometry was used to quantitate and compare the percentage of SA-βgal (-) cells in cultures four to six weeks following irradiation. At the time of harvest, 85.2 ± 1.6% of the cells in the irradiated cultures were SA-βgal (-), whereas 78.0 ± 2.6% of the cells in the unirradiated cultures were SA-βgal (-) (*P *< 0.001) (Figure 2B).

We next measured the expression of p16 protein by immunofluorescence in unirradiated and irradiated cultures harvested at identical times (Figure 2C). More than 20 random fields from unirradiated and irradiated B1450 and B1400 cultures were analyzed. Significantly more (70.5 ± 3.3% vs. 44.8 ± 3.5, *P *< 0.0001) cells were p16 (-) in the irradiated cultures than those in the unirradiated cultures (Figure 2D).

### The p16 gene is silenced epigenetically in the progeny of both unirradiated and irradiated HMEC

**Figure 3:**
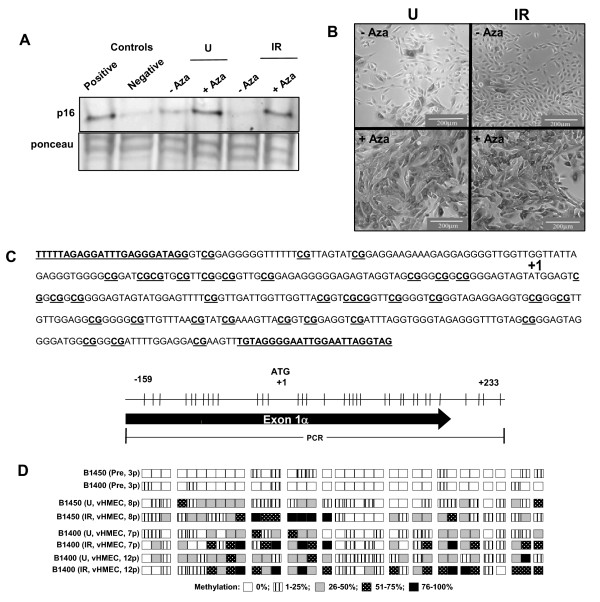
**The p16 gene is silenced epigenetically in both unirradiated and irradiated human mammary epithelial cells**. **(A) **Immunoblot analysis shows that p16 protein expression is very low in the eighth passage (*8p*) variant human mammary epithelial cells (vHMEC) cultures derived from both unirradiated and irradiated cells, but that it is readily induced to similar extents in both cultures treated with 5-aza-deoxycytidine (Aza). Lysates of HMEC undergoing stasis and MCF-7 cells were used as positive and negative controls, respectively. **(B) **p16 immunohistochemistry indicates that *8p *vHMEC cultures derived from both unirradiated and irradiated cells contain primarily p16(-) cells, and that p16 protein can be induced in virtually all cells in both cultures by 72 hr treatment with 3.3 μM 5-aza-deoxycytidine. **(C) **DNA sequence of the CpG island region and a map of the -159 to +233 region of the p16 gene indicate the 35 CpG sites (CG or vertical lines) analyzed for methylation status, and their positions relative to the translational start site (ATG) and exon 1α. Primers used for PCR amplification are underlined. **(D) **The percent methylation of specific CpG sites (indicated by degree of shading) in the -159 to +233 region of the p16 gene was determined by sequencing of individual bisulfite-treated DNAs obtained from the indicated pre-stasis HMEC and vHMEC cultures from specimens B1450 and B1400.

The lack of p16 expression in unirradiated vHMEC has been associated with methylation of the p16 gene promoter, as treatment with 5-aza-2'-deoxycytidine - an established inhibitor of DNA methylation, leads to induction of p16 expression and growth arrest in these cells [23-25]. To determine whether a qualitatively similar or distinct mechanism of p16 gene inactivation was responsible for the increased outgrowth of vHMEC in irradiated cultures, we first compared the susceptibility of irradiated and unirradiated cultures to 5-aza-2'-deoxycytidine-induced p16 expression. Immunoblot analyses and immunohistochemical staining showed a general lack of p16 protein expression in the cells that emerged from stasis cultures, although occasional p16 (+) cells continued to be observed in vHMEC cultures derived from both unirradiated and irradiated cells (Figure 3A and Figure 3B, upper panels). Treatment with 5-aza-2'-deoxycytidine induced the expression of p16 protein to a similar extent in virtually all cells derived from either unirradiated or irradiated cultures (Figure 3A and Figure 3B, lower panels), indicating that the increased outgrowth of vHMEC in irradiated cultures was due to reversible epigenetic silencing rather than to irreversible radiation-induced mutations.

To determine whether radiation resulted in qualitative and/or quantitative differences in the methylation of CpG sites in the p16 promoter, we used bisulfite sequencing to investigate the methylation status of 35 CpG sites spanning from -159 to +233, relative to the translation start site (+1), in the core CpG island region of the p16 gene in two specimens, B1450 and B1400, with and without irradiation (Figure 3C). The majority of the CpG sites were unmethylated in pre-stasis *3p *HMEC cultures harvested prior to stasis, whereas p16 promoter methylation was significantly increased in both unirradiated and irradiated vHMEC cultures, as indicated by the % methylation of individual CpG sites (Figure 3D). The methylation patterns were qualitatively and quantitatively distinct in irradiated and unirradiated cultures, however there was no discernable quantitative or qualitative correlation among p16 promoter methylation and passage level or radiation exposure status.

### Radiation of different doses and qualities promotes outgrowth of vHMEC

**Figure 4:**
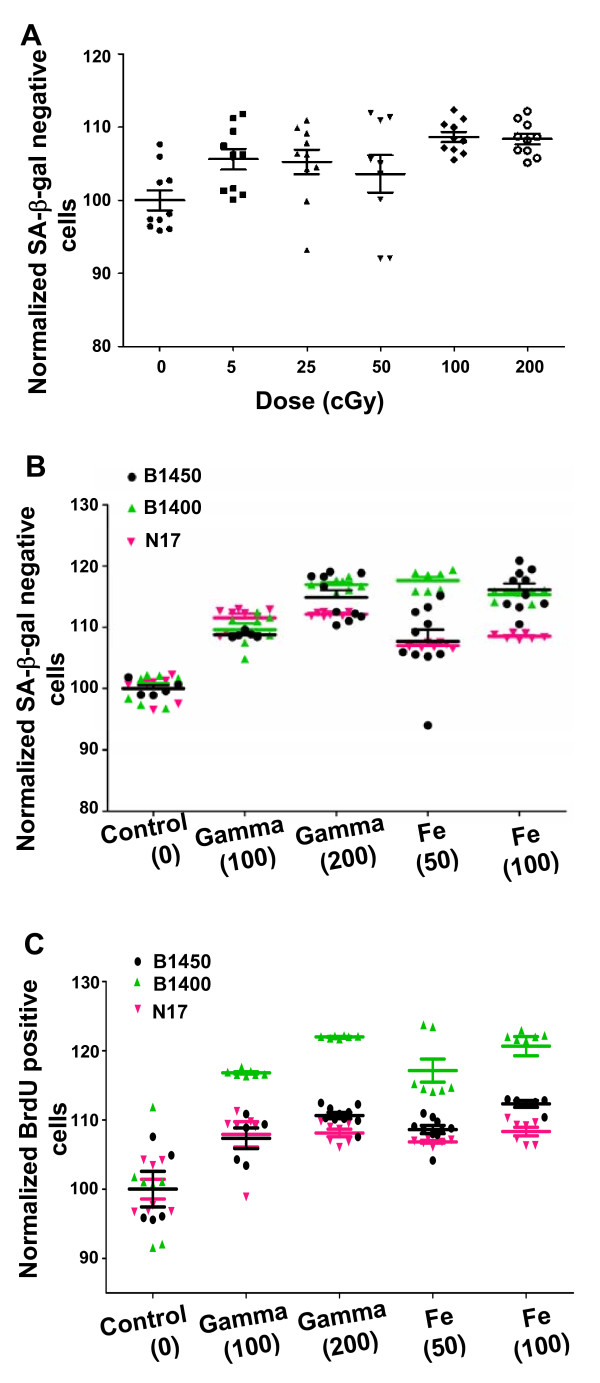
**Radiation of different doses and qualities promotes outgrowth of variant human mammary epithelial cells**. **(A) **Human mammary epithelial cells (HMEC) cultures (10 or more replicates per dose) from specimen B1450 were subjected to X-ray doses between 5 and 200 cGy. Each treatment group was normalized to a control group and the resulting means ± SE were plotted versus dose. (**B **and **C**) When assayed four to six weeks after irradiation with indicated doses (cGy) of ^56^Fe or γ-rays, indicated HMEC cultures showed statistically significant differences (*P *< 0.0001, non-parametric Wilcoxon test) in the percentages of (B) SA-βgal (-) and (C) BrdU (+) cells compared to control cultures. Scatter plots indicate the mean ± SE of between 5 and 10 independent replicates for three individual specimens assayed for each treatment.

To determine the dose dependence of the radiation-induced effect, replicate cultures from a single specimen (B1450) were exposed to X-ray doses of 0, 5, 25, 50, 100 and 200 cGy and progeny cultures were analyzed for SA-βgal activity four to six weeks following irradiation. Ten replicates were used for each treatment group, and the results for each treatment group were normalized to mean of an unirradiated group (Figure 4A). Cultures exposed to the highest doses (100 and 200 cGy) showed significant differences compared to unirradiated cultures (*P *< 0.0001; Wilcoxon/Kruskal-Wallis test). Cultures exposed to lower doses (5 and 25 cGy) showed similar trends, but the differences did not reach statistical significance. In addition, no significant linear correlation was observed between exposure dose and SA-βgal activity in the irradiated samples themselves, indicating the absence of a detectable dose-dependence in the dose range examined.

Densely ionizing ^56^Fe ions have been reported to be more potent inducers of genomic instability, cell transformation, and tumorigenesis than sparsely ionizing X-rays. The relative biological effectiveness (RBE) for cell killing of 1 GeV/amu ^56^Fe particles compared to X-rays was 1.80 at D_10 _(dose resulting in 10% survival by cell killing data) for HMEC [26]. RBE values reported for other endpoints such as tumorigenesis in mice have been much higher [27,28]. We investigated the effect of radiation quality on the outgrowth of vHMEC. The progeny of cells generated from three breast tissue specimens (B1450, B1400, N17) were irradiated with densely ionizing 1 GeV/amu ^56^Fe particles and compared to those irradiated with sparsely ionizing ^137^Cs γ-rays four to six weeks after exposure, using flow cytometry to measure SA-βgal activity and BrdU incorporation (Figure 4B and 4C) in 5 to 10 replicates from each specimen for each treatment. Although the response of individual specimens to radiation exposure varied, as expected, the cultures irradiated with ^137^Cs γ-rays contained significantly more SA-βgal (-) and BrdU (+) cells, as in the X-ray experiments, than unirradiated controls (*P *< 0.0001; Wilcoxon test). Notably, the responses to 1 GeV/amu ^56^Fe ions were comparable to those of γ-rays and were not dose dependent.

### Agent-based modeling can be used to accurately simulate HMEC growth kinetics

We considered two possible explanations for our observations that radiation promoted vHMEC outgrowth; first that radiation induced additional vHMEC and second, that radiation accelerated the selection of pre-existing vHMEC, consistent with studies by Holst *et al. *[29] demonstrating their existence in human breast tissues. To evaluate the likelihood of these alternative mechanisms, we defined underlying assumptions to use an agent-based model (ABM) to simulate vHMEC outgrowth.

ABMs are computer simulations that represent systems as collections of autonomous decision-making entities called agents [30]. Each agent is programmed to respond to situations using a set of contextual rules. These models are non-deterministic, are typically based on multiple simulations so that a range of behaviors can be established for a population, and have proven to be very useful in predicting emerging properties from complex systems [31-36]. Recently, ABMs have been used to predict the responses of cancer stem cell populations to ionizing radiation [37].

Based on experimental observations, we knew that the growth of HMEC is contact inhibited, that the area occupied by individual cells is reduced with increasing culture density, that all HMEC have limited replication capacity, that after a limited number of divisions most HMEC are capable of expressing p16 that initiates senescence, and that senescent cells do not readily replate following trypsinization. Thus we defined the following agent rules: 1) agents replicate as long as there is space surrounding them; 2) if there is no open space, agents can still replicate as smaller entities until reaching a minimum size; 3) when agents reach the maximum number of replications allowed, they stop dividing and their type changes permanently to a senescent type; 4) senescent agents do not reattach after detachment during subculture.

**Figure 5:**
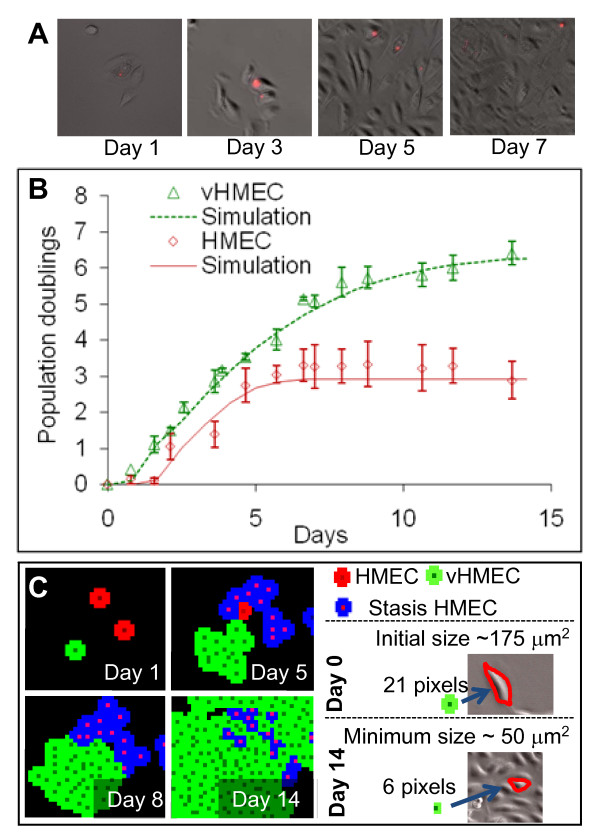
**The agent-based model is validated by experimental data**. (**A**) Growth characteristics of co-cultured human mammary epithelial cells (HMEC) (labeled with vital dye Qtracker565; Invitrogen) and variant human mammary epithelial cells (vHMEC) (unlabeled) were recorded by time-lapse microscopy. An example is presented of the changes in proportions of cells present in a field of view monitored over seven days. (**B**) Growth of co-cultured HMEC and vHMEC was compared with ABM simulations. Each data point represents the mean of four biological replicates. Pre-stasis HMEC (red diamonds) stopped proliferating after four days in culture in contrast to vHMEC (green triangles) that continued to proliferate until the cultures achieved confluence. Solid lines show agent-based simulations, based on the initial cell densities, cell cycle duration during the proliferative phase (26 hours for both cell types), maximum cell compression observed at full confluence, and an intrinsic limit on the number of population doublings achievable by HMEC. Note that the simulations of the model were well correlated with the experimental data, indicating that kinetic growth rates could be predicted accurately for both cell types using a limited number of assumptions. **(C) **Illustration of growth simulation. Normal growing HMEC (red) undergo limited divisions before losing proliferative potential and undergoing stasis (blue). vHMEC (green) are capable of unlimited of divisions in this example. At low density (Day 1), most cells occupy a maximum area (that is, 21 pixels equivalent to 175 μm^2^, matching light microscopy measurements). Cells continue dividing as space becomes more restricted (Day 5), until the available adjacent space reaches a minimum of six pixels (50 μm^2 ^at Day 14). Such simulations accurately recapitulate contact inhibition as measured by time-lapse microscopy.

To validate these rules, we first collected experimental data describing growth characteristics of co-cultured HMEC and vHMEC imaged by time-lapse microscopy (note: vHMEC were obtained by taking HMEC that had escaped stasis in a previous experiment). A representative series of time-lapse images shown in Figure 5A illustrates how vHMEC outgrew labeled HMEC over time, as vHMEC could not express p16 and thus would not undergo stasis. Figure 5B shows the means and standard deviations of population doublings measured experimentally for each cell type in four replicate experiments. Note that the HMEC used here were at a higher passage than in Figure 1, as they rapidly underwent stasis on Day 4 after having divided on average only three times. In contrast, vHMEC cells grew until they reached confluence. Thus, to simulate this series of co-culture experiments, a maximum limit of three divisions for HMEC agents was set, whereas no division limit for vHMEC agents was set. As described above, Figure 5C illustrates the key rule for contact inhibition, showing how agents stop growing once they have reached a minimum size determined experimentally by measuring the average area of fully confluent cells. The corresponding predictions from the ABM using these limited assumptions correlated well with the experimental data (Figure 5B), validating our rules.

**Figure 6:**
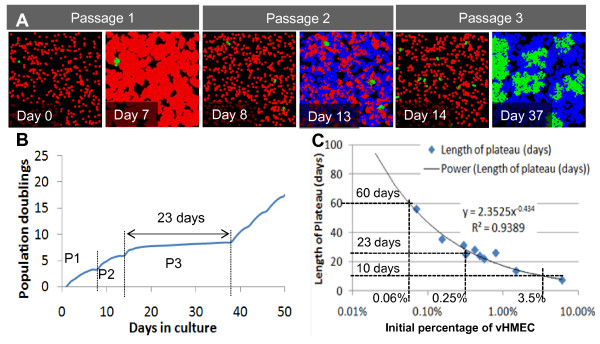
**Growth and senescence of heterogeneous human mammary epithelial cells cultures can be accurately modeled using the agent-based model**. (**A **and **B**) In a model extending over several passages, cells are initially plated at 20% confluence and replated when the populations reach 80% confluence. By Passage 3, most human mammary epithelial cells (HMEC) have undergone seven divisions, and enter stasis. As a result, the time to confluence is extended, and a population doubling plateau is observed. Ultimately, the variant human mammary epithelial cells (vHMEC) take up the available area, and exponential growth resumes. **(C) **Changing the initial density of vHMEC leads to variable plateau periods. This dependence is fitted by a power function that allows the calculation of the initial density of vHMEC present in the primary culture based on plateau length. The results of individual simulations are plotted as points.

Agent-based modeling was then used to simulate the repetitive subculture experiments reported in Figure 1. Based on the growth curves reported in Figure 1A (in contrast to agents used in the validation step), a maximum of seven divisions for HMEC agents was set. We assumed that vHMEC were present initially at low but unknown frequencies in primary cultures and allowed a maximum number of 40 divisions for vHMEC agents. Using these assumptions and experimentally derived parameters, simulations led to growth curves very similar to those observed in Figure 1A, with the same significant population doublings' plateaus observed when the majority of the cells entered stasis (that is, after seven population doublings when assuming that 0.25% of initial agents were vHMEC agents, as illustrated in Figure 6A and 6B). The plateau widths reflected the time taken by vHMEC agents to overgrow the senescent HMEC populations, and were inversely proportional to the fraction of vHMEC agents present in the initial populations; a relationship that could be fitted accurately by an inverse power function (Figure 6C). Assuming that the population doublings' plateau indicated the period of selection of a minority of vHMEC present in primary cultures, the model could then inform us of the initial proportions of vHMEC in real specimens by extrapolating the plateau widths measured in these specimens with the fit obtained in Figure 6C (that is, for the three unirradiated cultures profiled in Figure 1A, plateau durations of 23, 60, and 10 days corresponded to initial vHMEC proportions of 0.25, 0.06, and 3.5%, respectively). Anecdotally, we found that the initial percentage of vHMEC present in the primary culture derived from a reduction mammoplasty specimen (B1400) was similar to values found for reduction mammoplasties in an earlier report [29], but that the percentages of vHMEC in primary cultures derived from prophylactic mastectomies (B1450, B1389) were higher. Data from additional specimens of each type will be needed to determine whether this is a statistically significant correlation.

### Agent-based modeling predicts the accelerated outgrowth of the progeny of irradiated HMEC

**Figure 7:**
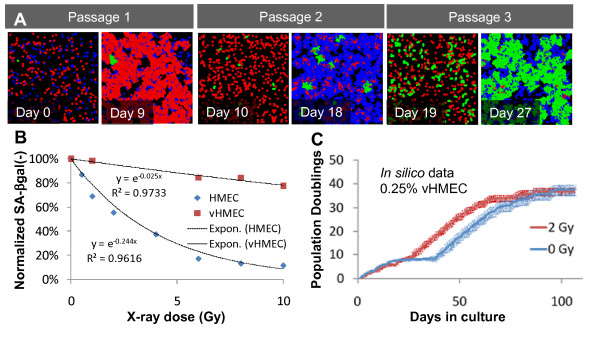
**Differential sensitivity to radiation-induced senescence and loss of growth leads to increased outgrowth of p16(-) human mammary epithelial cells during stasis**. **(A) **Time snapshots of one representative simulation of sequential subcultures executed using the same initial conditions as in Figure 6A, but including prematurely senescent human mammary epithelial cells (HMEC) agents (labeled blue) induced by exposure to 2 Gy of X-rays. Note the earlier and more robust outgrowth of vHMEC agents (labeled green) in this simulation versus the simulation shown in Figure 6A. **(B) **Growing pre- and post-stasis HMEC from the same individual were exposed to the indicated doses of X-rays, then stained and evaluated for SA-βgal expression. The resulting data were then normalized to the percentage of SA-βgal(-) HMEC in the respective unirradiated populations. Fitted curves were of the form (e^(-α.D)^) where D is the dose and α the fitted coefficient; R^2 ^values were >0.96 in both cases. These fitted curves were used to model radiation-induced senescence in the simulation depicted in (A). **(C) **Agent-based modeling was used to simulate the growth kinetics of HMEC cultures exposed to 0 or 2 Gy of X-rays. Data ± SE for five independent simulations for each condition are plotted. Note that the model predicts a shorter growth plateau in the irradiated sample, in accord with experimental observations.

Ionizing radiation is known to induce senescence in a variety of cell types, however susceptibility to radiation-induced senescence may vary with cell type. Therefore, to model the response of HMEC agents to ionizing radiation, we first had to experimentally measure the relative susceptibilities to radiation-induced senescence of pre-stasis (HMEC) and post-stasis (vHMEC) cells from the same individual. Measurement of SA-βgal levels indicated that normal HMEC were more likely to undergo senescence after the same dose of ionizing radiation than vHMEC; 6 Gy or more induced more than 90% SA-βgal (+) pre-stasis cells while even 10 Gy induced less than 20% SA-βgal (+) post-stasis cells (Figure 7B). Following 2 Gy of X-rays, the relative number of SA-βgal (+) cells increased more than 40% in pre-stasis populations, but less than 5% in post-stasis populations. The radiation-induced senescence parameters for each agent type were added to the ABM, and simulations were repeated. As illustrated in Figure 7A, the presence of senescent agents immediately in the first passage led to an earlier selection of vHMEC agents, and the preferential senescence of HMEC agents led to a proliferative advantage of vHMEC agents. Accordingly, the ABM kinetics predicted a shorter growth plateau after exposure to 2 Gy (Figure 7C), matching experimental observations.

## Discussion

A major challenge is to understand how cellular responses to radiation are integrated in a multicellular context to affect human health. In this study, we show how radiation can promote the outgrowth of pre-malignant cells by accelerating senescence of normal cells. We used ABM to show that the rapidity with which radiation-resistant vHMEC selectively populate cultures can be predicted by the initial proportion of the vHMEC present in primary cultures, the differential rate at which sensitive HMEC succumb to radiation-induced loss of growth potential, and the physical space available for expansion.

Together with p53, the p16 protein functions as a sentinel that integrates various cellular signals and stresses to limit proliferation. Methylation of the p16 gene has been identified *in situ *in histologically normal mammary epithelial cells of disease-free women [29]. A Luria-Delbrück fluctuation analysis suggests that such cells may be the source of vHMEC that continue to proliferate after most HMEC in long term cultures have undergone p16-associated stasis [29]. Expression of p16 appears to be strongly selected against in many human solid tumors, including those of the breast, where the gene encoding p16 is deleted in approximately 20% [38] and inactivated epigenetically in an additional 20% [39] of cases. Interestingly, exposure of workers in nuclear weapons manufacturing facilities to plutonium, for example, has been strongly linked (*P *= 0.03) to methylation of the p16 gene in lung adenocarcinomas [40]. Renal cell carcinomas from patients living in areas contaminated by the Chernobyl accident have also shown aberrant hypermethylation of the p16/p14 locus [41].

As we and others have observed, radiation itself does not appear to be a direct inducer of p16 (Additional file 1). Our new results indicate that radiation-induced stress can be integrated with p16-inducing factors that cause premature growth arrest and senescence. ABM simulations showed that radiation can advance the selection process, allowing vHMEC to overtake and fill the voids created by the prematurely senescing normal cells. A similar model, in which lesion growth is driven by opportunistic expansion of apoptosis-resistant p53 mutant cells, has recently been proposed for UVB-induced squamous cell carcinomas [42]. The expansion of such a variant population *in situ *would be expected to expand the target size in which additional malignancy promoting aberrations could occur, especially since this population has been shown to be particularly susceptible to genomic instability caused by telomere dysfunction and centrosome irregularities [6,13,14].

While accurately modeling the accelerated outgrowth of vHMEC from irradiated cultures, the current ABM does not explain why, in some cases, the total number of population doublings achieved by vHMEC from irradiated cultures exceeded that of vHMEC from unirradiated controls. This outcome could be due to either a radiation-induced increase in the number of vHMEC or modification of the phenotype of the existing vHMEC. Since the phenotypic feature of the vHMEC that allows them to avoid or overcome stasis is the stable repression of p16 expression, one possibility is that radiation directly affects epigenetic process(es) involved in the initiation or maintenance of this repression. DNA methylation-associated silencing of mammalian genes has been proposed to be a process, during which instances of spontaneous gene reactivation are initially frequent, becoming progressively less frequent over the course of multiple cell divisions [43,44]. In agreement with this hypothesis, recent experiments indicate that p16 gene methylation occurs progressively in clonal vHMEC populations after silencing has occurred [45]. Irradiation may lead to acceleration of this process and thus reduce the frequency of spontaneous p16 reactivation. Indeed, while occasional p16(+) cells could be observed in *8p *post-stasis cultures, they were more frequent in the progeny of unirradiated versus irradiated cultures (data not shown). The mechanism responsible for this difference remains to be discovered, but may involve radiation effects on sentinel proteins such as p53. Notably, radiation has been reported to relieve p53-mediated repression of DNA methyltransferase 1 (Dnmt1) in human colon carcinoma cells [46]. Under certain circumstances, Dnmt1, which has been shown to be continually required for p16 repression [47], will catalyze *de novo *methylation of specific promoter CpG islands [48]. Thus, radiation, acting through transient activation of p53, could cause *de novo *methylation and more stable silencing of the p16 gene.

## Conclusions

We have found that radiation can indirectly promote the outgrowth of a putative pre-malignant breast cell population by accelerating senescence of normal breast cells. Differential sensitivity of these cell populations to radiation-induced senescence has not previously been demonstrated. While the mechanism responsible for this differential sensitivity is beyond the scope of our present investigation, the work advances a systems-based paradigm of carcinogenic activity by a prototypic genotoxic agent, ionizing radiation. The ABM simulation of well-described HMEC phenotypes in primary cultures helped to distinguish between induced vHMEC and altered population kinetics as plausible explanations for the observed response to radiation. ABM showed that differential sensitivity to radiation-induced senescence can at least partly explain the enhanced outgrowth of vHMEC from irradiated cultures. This simple *in vitro *system illustrates the concept that heterogeneous responses of individual cells within a population must be integrated to achieve a system-level understanding of radiation effects.

## Abbreviations

ABM: agent-based model; FBS: fetal bovine serum; FDG: fluorescein digalactoside; HMEC: human mammary epithelial cells; LET: linear energy transfer; *p*: passage; p16: p16^INK4A^; PBS: phosphate buffer saline; RBE: relative biological effectiveness; SA-βgal: senescence associated β-galactosidase; v: variant.

## Competing interests

The authors declare that they have no competing interests.

## Authors' contributions

RM performed cell culture, molecular and cellular assays, participated in the design, analyzed and interpreted data, and helped to draft the manuscript. SC designed and performed imaging assays and agent-based modeling, analyzed and interpreted data, and helped to draft the manuscript. AB and WCH acquired tissue samples and established primary HMEC cultures, and WCH assisted with imaging assays. MHBH and PY initiated the studies, participated in the design and coordination of the study, helped analyze and interpret data, and helped to draft the manuscript. All authors have read and approved the manuscript.

## Supplementary Material

Additional file 1 **Supplementary Figure S1 - X-irradiation does not induce p16 directly**. p16 immunohistochemistry indicates that 3*p *HMEC cultures derived from specimen N17 did not express detectable p16 protein 24 hrs after irradiation with 2 Gy X-rays. Indicated negative and positive controls for specific antibody-dependent staining are shown in left panels. Click here for file
