# Supplementary material for: Promotion of variant human mammary epithelial cell outgrowth by ionizing radiation: an agent-based model supported by in vitro studies
Source: Breast Cancer Res. 2010 Feb 10;12(1):R11. doi: 10.1186/bcr2477 (PMC2880432; doi:10.1186/bcr2477)
Supplement: Additional file 1 — Supplementary Figure S1 - X-irradiation does not induce p16 directly. p16 immunohistochemistry indicates that 3p HMEC cultures derived from specimen N17 did not express detectable p16 protein 24 hrs after irradiation with 2 Gy X-rays. Indicated negative and positive controls for specific antibody-dependent staining are shown in left panels. [file bcr2477-S1.pdf]

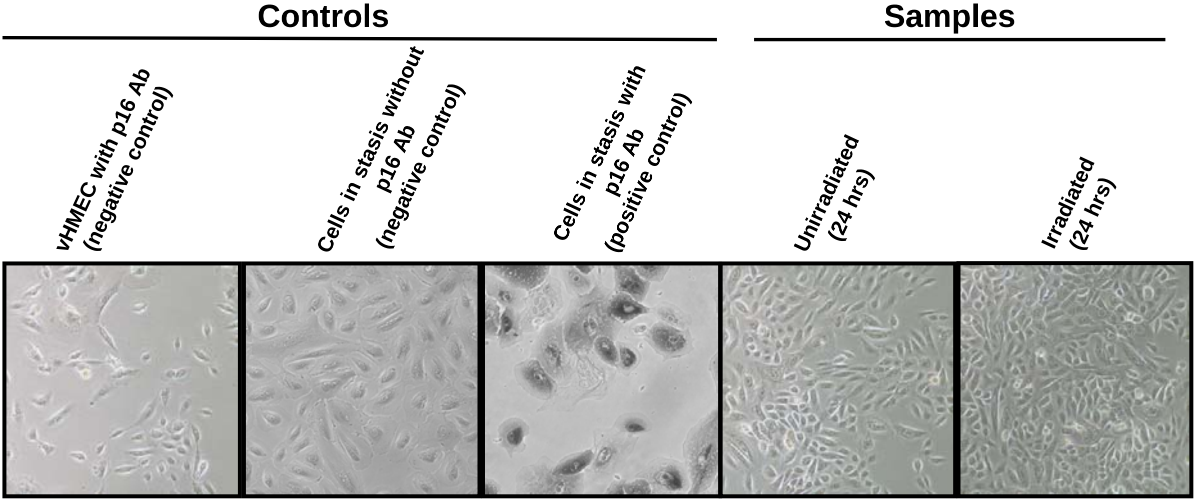

Controls
Samples
vHMEC with p16 Ab (negative control)
Cells in stasis without p16 Ab (negative control)
Cells in stasis with p16 Ab (positive control)
Unirradiated (24 hrs)
Irradiated (24 hrs)
